# Proposals for Paraphilic Disorders in the International Classification of Diseases and Related Health Problems, Eleventh Revision (ICD-11)

**DOI:** 10.1007/s10508-017-0944-2

**Published:** 2017-02-16

**Authors:** Richard B. Krueger, Geoffrey M. Reed, Michael B. First, Adele Marais, Eszter Kismodi, Peer Briken

**Affiliations:** 1 Department of Psychiatry, College of Physicians and Surgeons, New York State Psychiatric Institute, Columbia University, New York, NY USA; 2 Department of Psychiatry, New York Presbyterian Hospital, New York, NY USA; 3 Sexual Behavior Clinic, New York State Psychiatric Institute, 1051 Riverside Drive, Unit #45, New York, NY 10032 USA; 4 Department of Mental Health and Substance Abuse, World Health Organization, Geneva, Switzerland; 5 Global Mental Health Program, Columbia University Medical Center, New York, NY USA; 6 Department of Psychiatry and Mental Health, Groote Schur Hospital, University of Cape Town, Observatory, South Africa; 7 Geneva, Switzerland; 8 Global Health Justice Partnership, Yale Law School, New Haven, CT USA; 9 Institute for Sex Research and Forensic Psychiatry, University Medical Center Hamburg-Eppendorf, Hamburg, Germany

**Keywords:** Paraphilic disorders, ICD-11, Paraphilias, ICD-10, DSM-5, Disorders of sexual preference

## Abstract

The World Health Organization is currently developing the 11th revision of the International Classifications of Diseases and Related Health Problems (ICD-11), with approval of the ICD-11 by the World Health Assembly anticipated in 2018. The Working Group on the Classification of Sexual Disorders and Sexual Health (WGSDSH) was created and charged with reviewing and making recommendations for categories related to sexuality that are contained in the chapter of Mental and Behavioural Disorders in ICD-10 (World Health Organization 1992a). Among these categories was the ICD-10 grouping F65, Disorders of sexual preference, which describes conditions now widely referred to as Paraphilic Disorders. This article reviews the evidence base, rationale, and recommendations for the proposed revisions in this area for ICD-11 and compares them with DSM-5. The WGSDSH recommended that the grouping, Disorders of sexual preference, be renamed to Paraphilic Disorders and be limited to disorders that involve sexual arousal patterns that focus on non-consenting others or are associated with substantial distress or direct risk of injury or death. Consistent with this framework, the WGSDSH also recommended that the ICD-10 categories of Fetishism, Fetishistic Transvestism, and Sadomasochism be removed from the classification and new categories of Coercive Sexual Sadism Disorder, Frotteuristic Disorder, Other Paraphilic Disorder Involving Non-Consenting Individuals, and Other Paraphilic Disorder Involving Solitary Behaviour or Consenting Individuals be added. The WGSDSH’s proposals for Paraphilic Disorders in ICD-11 are based on the WHO’s role as a global public health agency and the ICD’s function as a public health reporting tool.

**Electronic supplementary material:**

The online version of this article (doi:10.1007/s10508-017-0944-2) contains supplementary material, which is available to authorized users.

## Introduction

The World Health Organization (WHO) is the global public health agency of the United Nations, whose mission is the attainment of the highest possible level of health by all people. The WHO’s core responsibilities, ratified by international treaty by the WHO’s 194 member states, include the development of international classification systems for health and the international standardization of diagnostic procedures (WHO, 2014). As an aspect of these responsibilities, the WHO is responsible for the International Classification of Diseases and Related Health Problems (ICD), currently in its tenth revision (ICD-10) (WHO, 1992b), which provides a mandatory framework for the WHO member states for the collection and reporting of health information. The WHO is currently revising the ICD, with approval of ICD-11 by the World Health Assembly expected in May 2018.

In addition to being the international standard for health information, the ICD is used by many member states as a framework for defining their responsibilities to provide free or subsidized health service to their citizens (International Advisory Group for the Revision of ICD-10 Mental and Behavioural Disorders, 2011). As an integral part of the global classification for all health conditions, the ICD chapter on Mental and Behavioural Disorders is by far the most widely used classification of mental disorders worldwide (Reed, Correia, Esparza, Saxena, & Maj, 2011). The current revision of the ICD—the first major revision in more than two decades—provides an important opportunity to improve the system, bringing it more in line with current evidence, practice, and human rights standards.

The aim of this article is to present the background, evidence base, and rationale for the proposed revisions to the ICD-10 grouping Disorders of sexual preference (F65), detailed diagnostic guidelines which are found in the *Clinical Descriptions and Diagnostic Guidelines* for ICD-10 Mental and Behavioural Disorders (WHO, 1992a). The WHO Department of Mental Health and Substance Abuse has technical responsibility for managing the activities involved in the current revision of the ICD-10 and in 2007 appointed an international advisory group to assist in this process. The advisory group has provided a description of the general principles underlying the development of the classification of Mental and Behavioural Disorders in ICD-11 (International Advisory Group for the Revision of ICD-10 Mental and Behavioural Disorders, 2011). With the consultation of the advisory group, a series of working groups was appointed to review available evidence, to develop proposals for changes to the ICD-10 Mental and Behavioural disorders categories, and to draft diagnostic guidelines for the categories within their area of responsibility. All working groups were required to be multidisciplinary and to include representation of all WHO global regions, including a substantial representation of low- and middle-income countries. A detailed description of the diagnostic guidance being developed by working groups has been provided (International Advisory Group for the Revision of ICD-10 Mental and Behavioural Disorders, 2011), and articles describing proposals in specific disorder areas have been published elsewhere (e.g., Drescher, Cohen-Kettenis, & Reed, 2016; Maercker et al., 2013; Reed et al. 2016a, b; Stein et al., 2016).

In relation to the ICD-10 Mental and Behavioural Disorder categories related to sexuality, sexual behavior, and gender identity, the WHO Department of Mental Health and Substance Abuse jointly appointed a Working Group on Sexual Disorders and Sexual Health (WGSDSH) with WHO Department of Reproductive Health and Research. This dual sponsorship was seen as important because of potentially overlapping areas of responsibility, knowledge, and expertise. The WGSDSH’s charge was informed by the public health mission of the WHO and the primary public health purpose of the ICD. In addition to providing the global standard for the collection and reporting of information about morbidity and mortality, many WHO member states use the ICD as a framework for defining their obligations to provide free or subsidized healthcare services to their citizens (International Advisory Group for the Revision of ICD-10 Mental and Behavioural Disorders, 2011). The WHO Department of Mental Health and Substance Abuse has indicated that developing a more effective tool for helping WHO member countries to reduce the disease burden associated with mental disorders is a central goal for ICD-11’s development and has identified improving the classification’s clinical utility and global applicability as critical means of achieving that goal.

The specific tasks of the WGSDSH included the review of available evidence and the development of proposals for modification of the categories, definitions, and guidelines for disorders related to sexual orientation, gender identity, sexual behaviors, and sexual dysfunctions that had been included in the chapter on Mental and Behavioural Disorders in the ICD-10. The WGSDSH was also asked to evaluate the proposals in this area for the American Psychiatric Association’s DSM-5 (American Psychiatric Association, 2013), at that time in preparation, and to examine their clinical utility and global applicability. The WGSDSH was further asked to draft diagnostic guidelines for the proposed categories in line with specifications provided by the WHO Department of Mental Health and Substance Abuse (First, Reed, Hyman, & Saxena, 2015).

In taking up its charge, the WGSDSH considered multiple sources of information. A literature search was completed in Ovid, MEDLINE, PubMed, PsychINFO, and Scopus from 1948 to the present. Relevant policy documents and related literature were provided by the World Health Organization, including the classifications of sexual disorders in ICD from ICD-6 (WHO, 1948) to the present. In particular, the Working Group reviewed the ICD-10 *Clinical Descriptions and Diagnostic Guidelines* in the relevant areas for the purposes of identifying problematic elements in terms of reliability, validity, and clinical utility that might need to be revised. In addition, submission of proposals for revisions to ICD-10 had been encouraged by WHO beginning in 2008 and could be submitted in three languages. Proposals relevant to the areas of the WGSDSH’s responsibility were received from a variety of scientific societies, professional associations, and advocacy organizations, as well as from several individual experts. In addition, proposals for the classification of sexual disorders and sexual health in ICD-11 were undertaken with awareness of the human rights standards endorsed by the United Nations.

Descriptions of proposals related to other areas of WGSDSH responsibility have been published elsewhere, including proposals related to the ICD-10 categories focused on sexual orientation (Cochran et al., 2014) and the ICD-10 categories focused on gender identity (Drescher et al., 2012, 2016). This article reviews the evidence base, rationale, and recommendations for Paraphilic Disorders categories in ICD-11. The proposals described in this article were originally developed by the WGSDSH and then were modified by the WGSDSH following international review and subsequent expert consultation conducted by WHO.^1^ The proposals described in this article will be tested in a series of field studies (Keeley et al., 2015) and will also be made available for review and comment (Reed et al., 2016a, b). WHO will revise the diagnostic guidelines based on the study results and the comments received prior to the anticipated approval of the ICD-11 by the World Health Assembly in 2018.

## History of Paraphilic Disorders in the ICD

The WHO was founded in 1948 and assumed responsibility for the revision and maintenance of the ICD as of that time as a part of its constitutional responsibilities. Editions of the ICD prior to the ICD-6 exclusively contained a classification of mortality (the first version was called “The International List of Causes of Death”) (WHO, 1993). It was not until the ICD-6 approved by the newly established World Health Assembly during the same year that the WHO was founded that a classification of morbidity, including mental disorders, was included in the classification. The ICD-6 chapter on Mental, Psychoneurotic, and Personality Disorders contained the category Sexual Deviation (WHO, 1948). Inclusion terms were used to designate specific phenomena that should be assigned to a particular category that do not have their own, separate categorical designation. In the ICD-6, exhibitionism, fetishism, pathologic sexuality, and sadism were listed as inclusion terms under the category Sexual Deviation. In the ICD-7, approved in 1955, this section of the classification was unchanged (WHO, 1955). The ICD-8, approved in 1965, ushered in a substantial expansion of categories related to paraphilias in the chapter on Mental Disorders. Under the grouping of Sexual Deviation, specific categories were included for Homosexuality, Fetishism, Paedophilia, Transvestitism, Exhibitionism, and Other Sexual Deviation. Masochism, narcissism, necrophilia, sadism, and voyeurism were listed as inclusion terms for Other Sexual Deviation (WHO, 1965).

The ICD-9, approved in 1975, included a grouping of Sexual Disorders and Deviation in the chapter on Mental Disorders (WHO, 1977).^2^ This grouping included specific categories for Homosexuality, Bestiality, Paedophilia, Transvestism, Exhibitionism, Trans-Sexualism, Disorders of Psychosexual Identity, Frigidity and Impotence, and Other Sexual Deviation or Disorder. Among the inclusion terms for Other Sexual Deviation or Disorder were fetishism, masochism, and sadism. The ICD-9 was the first version of the ICD classification to include definitions for each condition in the chapter on Mental Disorders. Prior to that, no definitions or other diagnostic guidance had been provided for any condition in the ICD.

Definitions for some categories related to paraphilias in the ICD-9 focused exclusively on specific sexual behaviors with no reference to arousal pattern. For example, Bestiality was defined as “Sexual or anal intercourse with animals” (WHO, 1977, p. 196), and Paedophilia was defined as “Sexual deviations in which an adult engages in sexual activity with a child of the same or opposite sex” (p. 196). However, for Exhibitionism, the idea of a preferential arousal pattern was introduced: “Sexual deviation in which the main sexual pleasure and gratification is derived from exposure of the genitals to a person of the opposite sex” (WHO, 1977, p. 197). The ICD-9 definition for Transvestism described this condition as being based on a specific arousal pattern and distinguished it from issues related to gender identity: “Sexual deviation in which sexual pleasure is derived from dressing in clothes of the opposite sex. There is no consistent attempt to take on the identity or behavior of the opposite sex” (WHO, 1977, p. 197).

The ICD-10—currently the official WHO classification of diseases and disorders—was approved in 1990 (WHO, 1992b). Disorders related to paraphilias were given their own grouping in the ICD-10 chapter on Mental and Behavioural Disorders, called Disorders of sexual preference (F65). This grouping included F65.0 Fetishism, F65.1 Fetishistic Transvestism, F65.2 Exhibitionism, F65.3 Voyeurism, F65.4 Paedophilia, F65.5 Sadomasochism, F65.6 Multiple Disorders of Sexual Preference, and F65.8 Other Disorders of Sexual Preference (World Health Organization, 1992a).^3^ Diagnostic requirements for these categories as provided in the Clinical Descriptions and Diagnostic Guidelines (CDDG) for ICD-10 Mental and Behavioural Disorders (World Health Organization, 1992a) are shown in Table 1.

**Table 1:** ICD-10 definitions and diagnostic guidelines for F65 Disorders of sexual preference (WHO, 1992a)

Diagnosis	Description and diagnostic guidelines
F65.0 Fetishism	Reliance on some non-living object as a stimulus for sexual arousal and sexual gratification. Many fetishes are extensions of the human body, such as articles of clothing or footware. Other common examples are characterized by some particular texture such as rubber, plastic, or leather. Fetish objects vary in their importance to the individual: in some cases they serve simply to enhance sexual excitement achieved in ordinary ways (e.g. having the partner wear a particular garment) *Diagnostic guidelines* Fetishism should be diagnosed only if the fetish is the most important source of sexual stimulation or essential for satisfactory sexual response Fetishistic fantasies are common, but they do not amount to a disorder unless they lead to rituals that are so compelling and unacceptable as to interfere with sexual intercourse and cause the individual distress Fetishism is limited almost exclusively to males
F65.1 Fetishistic transvestism	The wearing of clothes of the opposite sex principally to obtain sexual excitement *Diagnostic guidelines* The disorder is to be distinguished from simple fetishism in that the fetishistic articles of clothing are not only worn, but worn also to create the appearance of a person of the opposite sex. Usually more than one article is worn and often a complete outfit, plus wig and makeup. Fetishistic transvestism is distinguished from transsexual transvestism by its clear association with sexual arousal and the strong desire to remove the clothing once orgasm occurs and sexual arousal declines. A history of fetishistic transvestism is commonly reported as an earlier phase by transsexuals and probably represents a stage in the development of transsexualism in such cases *Includes*: Transvestic fetishism
F65.2 Exhibitionism	A recurrent or persistent tendency to expose the genitalia to strangers (usually of the opposite sex) or to people in public places, without inviting or intending closer contact. There is usually, but not invariably, sexual excitement at the time of the exposure and the act is commonly followed by masturbation. This tendency may be manifest only at times of emotional stress or crises, interspersed with long periods without such overt behaviour *Diagnostic guidelines* Exhibitionism is almost entirely limited to heterosexual males who expose to females, adult or adolescent, usually confronting them from a safe distance in some public place. For some, exhibitionism is their only sexual outlet, but others continue the habit simultaneously with an active sex life within long-standing relationships, although their urges may become more pressing at times of conflict in those relationships, although their urges may become more pressing at times of conflict in those relationships. Most exhibitionists find their urges difficult to control and ego-alien. If the witness appears shocked, frightened, or impressed, the exhibitionist’s excitement is often heightened
F65.3 Voyeurism	A recurrent or persistent tendency to look at people engaging in sexual or intimate behaviour such as undressing. This usually leads to sexual excitement and masturbation and is carried out without the observed people being aware
F65.4 Paedophilia	A sexual preference for children, usually of prepubertal or early pubertal age. Some paedophiles are attracted only to girls, others only to boys, and others again are interested in both sexes Paedophilia is rarely identified in women. Contacts between adults and sexually mature adolescents are socially disapproved, especially if the participants are of the same sex, but are not necessarily associated with paedophilia. An isolated incident, especially if the perpetrator is himself an adolescent, does not establish the presence of the persistent or predominant tendency required for the diagnosis. Included among paedophiles, however, are men who retain a preference for adult sex partners but, because they are chronically frustrated in achieving appropriate contacts, habitually turn to children as substitutes. Men who sexually molest their own prepubertal children occasionally approach other children as well, but in either case their behaviour is indicative of paedophilia
F65.5 Sadomasochism	A preference for sexual activity that involves bondage or the infliction of pain or humiliation. If the individual prefers to be the recipient of such stimulation this is called masochism; if the provider, sadism. Often an individual obtains sexual excitement from both sadistic and masochistic activities Mild degrees of sadomasochistic stimulation are commonly used to enhance otherwise normal sexual activity. This category should be used only if sadomasochistic activity is the most important source of stimulation or necessary for sexual gratification Sexual sadism is sometimes difficult to distinguish from cruelty in sexual situations or anger unrelated to eroticism. Where violence is necessary for erotic arousal, the diagnosis can be clearly established *Includes:* masochism, sadism
F65.6 Multiple Disorders of Sexual Preference	Sometimes more than one disorder of sexual preference occurs in one person and none has clear precedence. The most common combination is fetishism, transvestism, and sadomasochism
F65.8 Other Disorders of Sexual Preference	A variety of other patterns of sexual preference and activity may occur, each being relatively uncommon. These include such activities as making obscene telephone calls, rubbing up against people for sexual stimulation in crowded public places (frotteurism), sexual activity with animals, use of strangulation or anoxia for intensifying sexual excitement, and a preference for partners with some particular anatomical abnormality such as an amputated limb Erotic practices are too diverse and many too rare or idiosyncratic to justify a separate term for each. Swallowing urine, smearing feces, or piercing foreskin or nipples may be part of the behavioural repertoire in sadomasochism. Masturbatory rituals of various kinds are common, but the more extreme practices, such as the insertion of objects into the rectum or penile urethra, or partial self-strangulation, when they take the place of ordinary sexual contacts, amount to abnormalities. Necrophilia should also be coded here *Includes:* frotteurism, necrophilia
F65.9 Disorder of sexual preference, unspecified	*Includes:* sexual deviation NOS

## Considerations in Conceptualizing Paraphilic Disorders for ICD-11

Cochran et al. (2014) described several principles that were important to the WGSDSH’s consideration of the circumstances under which patterns of sexual arousal and sexual behavior might be conceptualized as mental disorders. The first principle is related to the ICD’s primary function as a global public health tool that provides the framework for international public health surveillance, health reporting, and the calculation of disease burden and disability. Many WHO member countries have also extended the uses of the ICD by using it as a framework for defining their obligations for defining free or subsidized treatment (International Advisory Group for the Revision of ICD-10 Mental and Behavioural Disorders, 2011). In the context of Paraphilic Disorders, it is of central relevance from WHO’s perspective to distinguish conditions that are relevant to public health and indicate the need for health services from those that are merely descriptions of private behaviors that do not have an appreciable public health impact and for which treatment is neither indicated not sought. The ICD-10 classification of Disorders of sexual preference, which in many cases merely describes the sexual behavior involved (e.g., “The wearing of clothes of the opposite sex principally to obtain sexual excitement”), did not address the issue of their public health relevance. A complex consideration of under what circumstances atypical sexual behaviors represent conditions of public health significance and clinical importance has been perhaps the most important driver of proposed changes for the ICD-11 classification of Paraphilic Disorders.

Second, there are a variety of circumstances under which individuals may seek or may benefit from mental health services that do not represent disorders or diseases. The ICD-10 recognized this through the inclusion of a set of categories referred to as “Factors Influencing Health Status and Encounters with Health Services.” The ICD-10 described the use of these categories as appropriate “when a person who may or may not be sick encounters the health services for some specific purpose, such as to receive limited care…or to discuss a problem which is in itself not a disease or injury” (World Health Organization, 1992b, p. 1125). The corresponding proposed chapter in ICD-11 includes categories for “Counseling related to sexuality,” which may include health services provided related to issues of sexual knowledge, sexual attitudes, sexual behavior, and sexual relationships that are not considered to represent disorders. That is, it is not necessary to diagnose a disorder simply to indicate a need for counseling or information related to sexuality and, conversely, a perceived need for this type of intervention does not automatically indicate the presence of a disorder.

A third critical issue when considering how Paraphilic Disorders should be conceptualized is ICD-10’s explicit statement that “Social deviance or conflict alone, without personal dysfunction, should not be included in mental disorder as defined here” (World Health Organization, 1992a, p. 5). Cochran et al. pointed out that a variety of factors related to social environmental stressors and cultural norms related to sexuality (e.g., stigmatization, rejection, isolation, and criminalization) can have profound impacts on psychological experiences and behaviors that do not necessarily reflect an underlying sexual disorder. “In addition, social or political disapproval has resulted at times in abuse of diagnoses—especially psychiatric diagnoses—to harass, silence, or imprison persons whose behavior violates social norms or challenges existing authority structures” (p. 674). If a pattern of behaviors has no importance in terms of public health surveillance and reporting and does not have clinical importance in indicating a need for treatment or its association with distress or functional impairment, then the basis for defining that behavior pattern as a disease entity is highly questionable and may serve primarily to convey social judgment about that behavior. Cochran et al. concluded that the social deviance exclusion was critically important in considering this issue: “If a disease label is to be attached to a social condition, it is essential that it has a demonstrable clinical utility, for example, by identifying a legitimate mental health need, and its use should not exacerbate existing stigma, violence and discrimination” (p. 674).

## Review of Literature

Literature searches for articles using the terms paraphilias or Disorders of sexual preference and the international classification of diseases were conducted and yielded only a small number of articles, most of which were not relevant. Given the relevance of articles about paraphilias in the diagnostic and statistical manuals of the American Psychiatric Association, these were also reviewed. Among the articles so identified, Gayford (1997) reviewed the ICD-10 Disorders of sexual preference and the DSM paraphilias and concluded that, “To consider all Disorders of sexual preferences as equally pathological, or to tar all paraphiliacs with the same brush, is unfair and misleading. Harmless pleasure, dangerous activity and abuse of others are aspects that have to be evaluated, taking into consideration both the legal and moral dimensions” (p. 313). Ahlers, Schaefer, and Beier (2006) compared the sexual disorder diagnoses in DSM-IV and ICD-10 and concluded that DSM-IV was more precise and that some disorders were not named despite their clinical relevance and suggested that improvement was needed in the classification system. Berner, Berger, and Hill (2003) reviewed sexual sadism in ICD-10 and DSM-IV and noted that sadomasochism was combined in ICD-10 and separated into sadism and masochism in DSM-IV. They concluded that sexual sadism (but not masochism) was an important risk factor for sexual offending. Berner and Briken (2007) reviewed diagnoses in DSM-IV and ICD-10 and noted that paraphilic symptoms sometimes progressed to obsessive or addictive forms associated with loss of self-control, but could also occur as single incidents or as episodic events.

Reiersøl and Skeid (2006) argued that the three diagnostic categories in ICD-10: Fetishism (F65.0), Fetishistic Transvestism (F65.1), and Sadomasochism (F65.5), should not be considered illnesses and should be removed from the ICD. They argued that these disorders involved behaviors that were consensual and did not involve harm to self or others, pointing out that, in ICD-10, an individual can be diagnosed with these disorders solely because they practice the relevant behavior, without regard to its health or mental health consequences or associated distress and disability. Reiersøl and Skeid further argued that these diagnoses represented the stigmatization of socially atypical behavior and of the individuals with such sexual interests and did not meet the requirements for being considered a mental disorder. They further suggested that distress or shame that individuals experience related to their sexual preference might grow out of societal disapproval rather than representing an integral aspect of the sexual preference itself. This suggestion is consistent with previous research on the minority stress model among lesbian, gay, and bisexual populations (Meyer, 2003).

Laws governing sadomasochistic activities in some countries have been challenged (Bennett, 2013; Green, 2001), and the United Nations had called upon member states to ensure that individuals can freely express their sexuality (United Nations High Commissioner for Human Rights, 2011; World Health Organization, 2015). According to the Nordic Centre for Classifications in Health Care, the WHO Collaborating Center for classifications that comprises the government health statistics agencies for these countries, several Scandinavian countries have been responsive to these issues by modifying their national lists of officially accepted ICD-10 diagnoses by removing several categories from the Disorder of sexual preference (F65) grouping (Nordic Centre for Classifications in Health Care, 2015). Denmark removed the category Sadomasochism in 1995. In 2009, Sweden removed the categories Fetishism, Fetishistic Transvestism, Sadomasochism, and Multiple Disorders of Sexual Preference, and these same categories were removed by Norway in 2010 and by Finland in 2011. The unusual step of countries removing ICD diagnoses from their national classifications clearly constitutes a criticism of their inclusion in a diagnostic manual of mental disorders.

## Major Recommendations and Discussion

The following sections summarize the major changes proposed by the WGSDSH for the ICD-11 classification of Paraphilic Disorders, as compared to the ICD-10 classification of Disorders of sexual preference and the rationale for the recommended changes.

### Renaming Section F65 Disorders of Sexual Preference to Paraphilic Disorders and Overall Definition

The WGSDSH has recommended that a new section named Paraphilic Disorders replace the current ICD-10 section F65, Disorders of sexual preference. This new term better represents the content of this section, which includes entities that involve atypical sexual interests and which additionally meet the general definition of a mental disorder (International Advisory Group for the Revision of ICD-10 Mental and Behavioural Disorders, 2011). That is, the mere fact that an individual has an “atypical” pattern of sexual arousal in the sense that it differs from what may be arousing to most other people or from what would be considered normative in a given culture or subculture does not indicate that the individual has a mental disorder.

In defining what constitutes a Paraphilic Disorder, the WGSDSH considered that simply naming or describing specific sexual interest or behaviors, as in ICD-10, was not sufficient as a basis for definitions and diagnostic guidelines for Paraphilic Disorders in ICD-11. Specific sexual behaviors may occur for a variety of reasons; the WGSDSH has proposed that the diagnostic requirements for Paraphilic Disorders include an underlying pattern of persistent and intense atypical sexual arousal, manifested by sexual thoughts, fantasies, urges, and/or behaviors. Moreover, the WGSDSH considered which types of atypical sexual arousal patterns should be considered to be important from WHO’s perspective as a public health agency and, through their inclusion in the ICD-11, should be designated as appropriate targets for health services and public health reporting. The WGSDSH has recommended inclusion in the ICD-11 of arousal patterns whose focus involves others whose age or status renders them unwilling or unable to consent (e.g., prepubertal children, an unsuspecting individual being viewed through a window, an animal), in which the individual has acted on the arousal pattern or is markedly distressed by it. In addition, arousal patterns that do involve consenting adults or solitary behaviors should be diagnosable as Paraphilic Disorders when: (1) the individual is markedly distressed by the nature of the arousal pattern and the distress is not simply a consequence of rejection or feared rejection of the arousal pattern by others or (2) the nature of the paraphilic behavior involves significant risk of injury or death. The proposed general definition for Paraphilic Disorders (World Health Organization, 2016) is given in Table 2.

**Table 2:** ICD-11 proposed diagnostic guidelines for Paraphilic Disorders

***Paraphilic Disorders, General Definition***
Paraphilic disorders are characterized by persistent and intense patterns of atypical sexual arousal, manifested by sexual thoughts, fantasies, urges, or behaviours, the focus of which involves others whose age or status renders them unwilling or unable to consent and on which the person has acted or by which he or she is markedly distressed. Paraphilic disorders may include arousal patterns involving solitary behaviours or consenting individuals only when these are associated with marked distress that is not simply a result of rejection or feared rejection of the arousal pattern by others or with significant risk of injury or death
***Diagnostic Guidelines for Paraphilic Disorders***
**Exhibitionistic Disorder** *Essential (Required) Features*: A sustained, focused and intense pattern of sexual arousal—as manifested by persistent sexual thoughts, fantasies, urges, or behaviours—that involves exposing one’s genitals to an unsuspecting person in public places, usually without inviting or intending closer contact The individual must have acted on these thoughts, fantasies or urges or be markedly distressed by them *Boundary with Other Disorders and Normality*: By definition, Exhibitionistic Disorder specifically excludes consensual exhibitionistic behaviours that occur with the consent of the person or persons involved. Moreover, in some cultures there are socially sanctioned forms of public nudity, which do not constitute Exhibitionistic Disorder. (*Boundary with normality*) The occurrence or a history of behaviours involving exposing oneself to non-consenting individuals is insufficient to establish a diagnosis of Exhibitionistic disorder. Rather, these behaviours must reflect a sustained, focused, and intense pattern of sexual arousal. When this is not the case, other causes of the behaviour need to be considered. For example, exhibitionistic behaviours that do not reflect an underlying, persistent pattern of sexual arousal may occur in the context of some mental and behavioural disorders, such as manic episodes or dementia, or in the context of substance intoxication. (*Boundary with other mental and behavioural disorders, including substance intoxication)* Many sexual crimes involving exposing oneself in public may simply reflect actions or behaviours that are not associated with a sustained paraphilic underlying arousal pattern. Rather, these behaviours may be transient and occur impulsively or opportunistically. The diagnosis of Exhibitionistic disorder requires that these behaviours be a manifestation of a sustained, focused, and intense pattern of sexual arousal. (*Boundary with sexual crimes that do not involve a Paraphilic Disorder*)
**Voyeuristic Disorder** *Essential (Required) Features*: A sustained, focused and intense pattern of sexual arousal—as manifested by persistent sexual thoughts, fantasies, urges, or behaviours—that involves stimuli such as observing an unsuspecting person who is naked, in the process of disrobing, or engaging in sexual activity The individual must have acted on these thoughts, fantasies or urges or be markedly distressed by them *Boundary with Other Disorders and Normality*: By definition, Voyeuristic Disorder specifically excludes consensual voyeuristic behaviours that occur with the consent of the person or persons being observed. (*Boundary with normality*) The occurrence or a history of behaviours involving observing an unsuspecting individual who is naked, in the process of disrobing, or engaging in sexual activity is insufficient to establish a diagnosis of Voyeuristic Disorder. Rather, these behaviours must reflect a sustained, focused, and intense pattern of sexual arousal. When this is not the case, other causes of the behaviour need to be considered. For example, voyeuristic behaviours that do not reflect an underlying, persistent pattern of sexual arousal may occur in the context of some mental and behavioural disorders, such as manic episodes or dementia, or in the context of substance intoxication. (*Boundary with other mental and behavioural disorders, including substance intoxication)* Many sexual crimes involving observing non-consenting or unwilling others may simply reflect actions or behaviours that are not associated with a sustained underlying paraphilic arousal pattern. Rather, these behaviours may be transient and occur impulsively or opportunistically. The diagnosis of Voyeuristic Disorder requires that these behaviours be a manifestation of a sustained, focused, and intense pattern of sexual arousal. (*Boundary with sexual crimes that do not involve a Paraphilic Disorder)* *Additional Features*: The act of observing is for the purpose of achieving sexual excitement and does not necessarily involve an attempt to initiate sexual activity with the person being observed. Orgasm by masturbation may occur during the voyeuristic activity or later in response to memories of what the individual has seen. More recently, so-called ‘video voyeurs’ have been described who use video equipment to record individuals in public or private places where there is an expectation of privacy
**Pedophilic Disorder** *Essential (Required) Features*: A sustained, focused, and intense pattern of sexual arousal—as manifested by persistent sexual thoughts, fantasies, urges, or behaviours—involving pre-pubertal children The individual has acted on these thoughts, fantasies or urges or be markedly distressed by them *Boundary with Other Disorders and Normality*: A broad range of sexual behaviour with peers may occur in children or adolescents. This diagnosis does not apply to sexual behaviours among pre- or post-pubertal children with peers who are close in age. (*Boundary with normality*) The occurrence or a history of sexual behaviours involving pre-pubertal children is insufficient to establish a diagnosis of Pedophilic Disorder. Rather, these behaviours must reflect a sustained, focused, and intense pattern of pedophilic sexual arousal. When this is not the case, other causes of the behaviour need to be considered. For example, sexual behaviours involving children that do not reflect an underlying, persistent pattern of pedophilic sexual arousal may occur in the context of some mental and behavioural disorders, such as manic episodes or dementia, or in the context of substance intoxication. (*Boundary with other mental and behavioural disorders, including substance intoxication)* Many sexual crimes involving pre-pubertal children are not associated with an underlying, persistent pattern of pedophilic sexual arousal. Rather, these behaviours may be transient and occur impulsively or opportunistically. The diagnosis of Pedophilic Disorder requires that sexual behaviour involving pre-pubertal children be a manifestation of a sustained, focused, and intense pattern of pedophilic sexual arousal. (*Boundary with sexual crimes that do not involve a Paraphilic Disorder*) Some adolescents present with a history of sexually abusing younger children. The diagnosis of Pedophilic Disorder should be applied with outmost caution to adolescents. Unless there is a persistent pattern of such behaviour, reflecting a sustained, focused, and intense pattern of sexual arousal focused on pre-pubertal children, the diagnosis of Pedophilic Disorder is inappropriate. (*Boundary with sexually aggressive behaviour in adolescents)* *Additional Features*: Some individuals with Pedophilic Disorder are attracted only to males, others only to females, and others to both Some individuals act on their pedophilic urges only with family members, while others have victims outside their immediate family or both
**Coercive Sexual Sadism Disorder** *Essential (Required) Features*: A sustained, focused and intense pattern of sexual arousal—as manifested by persistent sexual thoughts, fantasies, urges or behaviours—that involves the infliction of physical or psychological suffering on a non-consenting person The individual must have acted on these thoughts, fantasies or urges or be markedly distressed by them *Boundary with Other Disorders and Normality*: By definition, Coercive Sexual Sadism Disorder specifically excludes consensual sexual sadism and masochism. (*Boundary with normality*) The occurrence or a history of sexual behaviours involving the infliction of physical or psychological suffering on non-consenting individuals is insufficient to establish a diagnosis of Coercive Sexual Sadism Disorder. Rather, these behaviours must reflect a sustained, focused, and intense pattern of coercive sexual sadistic arousal. When this is not the case, other causes of the behaviour need to be considered. For example, occasionally, sexual behaviours involving the infliction of physical or psychological suffering on non-consenting individuals may occur in the context of a manic episode or while the individual is under the influence of substances, particularly stimulants, when this does not reflect an underlying, persistent pattern of sexual arousal. (*Boundary with other mental and behavioural disorders, including substance intoxication)* Many sexual crimes involving non-consenting individuals who experience physical or psychological suffering as a result of the sexual crime are not associated with an underlying, persistent pattern of sexual arousal. Rather, these behaviours may be transient and occur impulsively or opportunistically. The diagnosis of Coercive Sexual Sadism Disorder requires that sexual behaviour involving the infliction of physical or psychological suffering on non-consenting individuals be a manifestation of a sustained, focused, and intense pattern of sexual arousal. (*Boundary with sexual crimes that do not involve a Paraphilic Disorder)* Conduct-Dissocial Disorder is characterized by a pervasive pattern of disregard for and violation of the rights of others. Coercive or sadistic sexual behaviours that occur in the context of Conduct-Dissocial Disorder but that do not reflect an underlying, persistent pattern of sexual arousal involving the infliction of physical or psychological suffering should not be used as a basis for diagnosing Coercive Sexual Sadism Disorder. In cases in which the diagnostic requirements of both disorders are met, both diagnoses may be assigned. (*Boundary with Conduct*-*Dissocial Disorder*)
**Frotteuristic Disorder** *Essential (Required) Features*: A sustained, focused and intense pattern of sexual arousal—as manifested by persistent sexual thoughts, fantasies, urges, or behaviours—that involves touching or rubbing against a non-consenting person in public places The individual must have acted on these thoughts, fantasies or urges or be markedly distressed by them *Boundary with Other Disorders and Normality*: By definition, Frotteuristic Disorder specifically excludes consensual touching or rubbing that occur with the consent of the person or persons involved. (*Boundary with normality*) The occurrence or a history of behaviours involving sexual touching or rubbing against non-consenting individuals in public places is insufficient to establish a diagnosis of Frotteuristic Disorder. Rather, these behaviours must reflect a sustained, focused, and intense pattern of frotteuristic sexual arousal. When this is not the case, other causes of the behaviour need to be considered. For example, inappropriate touching or rubbing against others that does not reflect an underlying, persistent pattern of sexual arousal may occur in the context of some mental and behavioural disorders, such as manic episodes or dementia, or in the context of substance intoxication. (*Boundary with other mental and behavioural disorders, including substance intoxication)* Many sexual crimes involving inappropriate touching or rubbing against others are not associated with an underlying, persistent pattern of paraphilic sexual arousal. Rather, these behaviours may be transient and occur impulsively or opportunistically. The diagnosis of Frotteuristic disorder requires that sexual touching or rubbing behaviours be a manifestation of a sustained, focused, and intense pattern of sexual arousal. (*Boundary with sexual crimes that do not involve a Paraphilic Disorde*r)
**Other Paraphilic Disorder Involving Non-Consenting Individuals** *Essential (Required) Features*: A sustained, focused and intense pattern of atypical sexual arousal, as manifested by sexual thoughts, fantasies, urges, and/or behaviours, in which the focus of the arousal pattern involves others whose age or status renders them unwilling or unable to consent that is not specifically described in any of the other named Paraphilic Disorders categories (e.g., arousal patterns involving corpses or animals) The individual must have acted on these thoughts, fantasies or urges or be markedly distressed by them The presentation does not satisfy the diagnostic requirements of Coercive sexual sadism disorder, Pedophilic disorder, Voyeuristic disorder, Exhibitionistic disorder, or Frotteuristic disorder *Boundary with Other Disorders and Normality*: Other Paraphilic Disorder Involving Non-Consenting Individuals specifically excludes sexual behaviours that occur with the consent of the person or persons involved, provided that they are by age and status able to provide such consent. (*Boundary with normality*) The occurrence or a history of sexual behaviours involving others whose age or status renders them unwilling or unable to consent is insufficient to establish a diagnosis of Other Paraphilic Disorder Involving Non-Consenting Individuals. Rather, these sexual behaviours must reflect a sustained, focused, and intense pattern of paraphilic sexual arousal. When this is not the case, other causes of the sexual behaviour need to be considered. For example, sexual behaviours involving non-consenting individuals that do not reflect an underlying, persistent pattern of sexual arousal may occur in the context of some mental and behavioural disorders, such as manic episodes or dementia, or in the context of substance intoxication. (*Boundary with other mental and behavioural disorders, including substance intoxication)* Many sexual crimes involving non-consenting individuals may simply reflect actions or behaviours that are not associated with a sustained underlying paraphilic arousal pattern. Rather, these behaviours may be transient and occur impulsively or opportunistically. The diagnosis of Other Paraphilic Disorder Involving Non-Consenting Individuals requires that these behaviours be a manifestation of a sustained, focused, and intense pattern of paraphilic sexual arousal. (*Boundary with sexual crimes that do not involve a Paraphilic Disorder*)
**Paraphilic Disorder Involving Solitary Behaviour or Consenting Individuals** *Essential (Required) Features*: A sustained, focused and intense pattern of atypical sexual arousal, as manifested by sexual thoughts, fantasies, urges, and/or behaviours that involves consenting adults or solitary behaviour One of the following two elements must be present: (1) The person is markedly distressed by the nature of the arousal pattern and the distress is not simply a consequence of rejection or feared rejection of the arousal pattern by others; or (2) The nature of the paraphilic behaviour involves significant risk of injury or death either to the individual (e.g., asphyxophilia or achieving sexual arousal by restriction of breathing) or to the partner (e.g., consensual sadism that results in injuries requiring medical treatment) If the diagnosis is assigned based on significant risk of injury or death, this risk should be directly and immediately connected to the paraphilic behaviour. For example, a presumed risk of increased exposure to sexually transmitted infections is not a sufficient basis for assigning this diagnosis *Boundary with Other Disorders and Normality*: The fact that an individual’s pattern of sexual arousal deviates from social or cultural norms is not a basis for assigning this diagnosis. An arousal pattern that involves consenting adults or solitary behaviour and that is not associated with marked distress that is not simply a consequence of rejection or feared rejection of the arousal pattern by others or with a significant risk of injury or death is not considered a disorder. (*Boundary with normality*) The occurrence or a history of atypical sexual behaviours is insufficient to establish a diagnosis of Paraphilic Disorder Involving Solitary Behaviour or Consenting Individuals. Some atypical sexual behaviours may occur impulsively or opportunistically or as a means of personal and sexual exploration and are not associated with a sustained underlying arousal pattern. The diagnosis of Paraphilic Disorder Involving Solitary Behaviour or Consenting Individuals requires that these behaviours be a manifestation of a sustained, focused, and intense pattern of paraphilic sexual arousal, in addition to distress or significant risk of injury or death. (*Boundary with normality*) When distress related to an arousal pattern involving consenting adults or solitary behaviour is entirely attributable to rejection or feared rejection of the arousal pattern by others (e.g., a partner, family, society), a diagnosis of Paraphilic Disorder Involving Solitary Behaviour or Consenting Individuals should not be assigned. Instead, codes related to counseling interventions from the chapter on Factors Influencing Health Status and Contact with Health Services may be considered. These include ‘Counseling related to sexual knowledge and sexual attitude’, ‘Counseling related to sexual behaviour and sexual relationships of the patient’, and ‘Counseling related to sexual behaviour and sexual relationship of couple’. *(Boundary with normality and with counseling related to sexual knowledge, attitudes, behaviour, and relationships)* If distress related to rejection or feared rejection of the arousal pattern by others has reached a point that presenting symptoms meet the diagnostic requirements for another mental disorder (e.g., Adjustment Disorder, a Depressive Disorder, an Anxiety Disorder), then that diagnosis should be assigned (rather than Paraphilic Disorder Involving Solitary Behaviour or Consenting Individuals). (*Boundary with other mental and behavioural disorders)* This diagnosis should not be applied to individuals who are distressed about homosexual or bisexual sexual orientation. If an individual is presenting for treatment based on such distress, codes related to counseling interventions from the chapter on Factors Influencing Health Status and Contact with Health Services may be considered. These include ‘Counseling related to sexual knowledge and sexual attitude’, ‘Counseling related to sexual behaviour and sexual relationships of the patient’, and ‘Counseling related to sexual behaviour and sexual relationship of couple’. If the pattern of distress-related symptoms meets the definitional requirements for another mental disorder (e.g., Adjustment Disorder, a Depressive Disorder, an Anxiety Disorder), then that diagnosis should be assigned. *(Boundary with distress related to sexual orientation)* Sexual behaviours that are atypical for the individual that do not reflect an underlying, persistent pattern of sexual arousal may occur in the context of some mental and behavioural disorders, such as manic episodes or dementia, or in the context of substance intoxication. If the sexual behaviours involved do not reflect an underlying, persistent pattern of sexual arousal, a diagnosis of Paraphilic Disorder Involving Solitary Behaviour or Consenting Individuals should not be assigned. *(Boundary with other mental and behavioural disorders, including substance intoxication)*

### Deletion of F65.0 Fetishism, F65.1 Fetishistic Transvestism, F65.5 Sadomasochism, and F65.6 Multiple Disorder of Sexual Preference

The WGSDSH has recommended the removal of the three of the named diagnostic categories currently included in ICD-10 Disorders of sexual preference (F65) from the ICD-11: Fetishism, Fetishistic Transvestism, and Sadomasochism. These conditions involved consensual or solitary sexual activity that do not involve inherent harm to self or others and are not necessarily distressing to the individual or associated with functional impairment. Therefore, the WGSDSH did not consider these arousal patterns per se to represent mental disorders or to be an appropriate focus of public health surveillance and reporting, but more accurately as variants in sexual arousal. The inclusion of these diagnoses can therefore be seen as inconsistent with human rights principles endorsed by the UN and the WHO (Drew et al., 2011) by stigmatizing those individuals practicing such behavior without clinical or public health benefit. There was no justification for maintaining an ostensible requirement that WHO member states collect statistics on these conditions and report on them to WHO. According to current ICD-11 proposals (see sections below), cases in which these arousal patterns are associated with marked distress or significant risk of injury or death could be accommodated under other categories in the ICD-11. The category of Multiple Disorders of Sexual Preference was also recommended for deletion because it is not clinically informative. Instead, if an individual meets the diagnostic requirements for more than one Paraphilic Disorder in ICD-11, multiple diagnoses may be assigned. This is consistent with the diagnostic conventions used with other Mental and Behavioural Disorders and throughout the ICD-11 classification.

### Inclusion of Exhibitionistic Disorder, Voyeuristic Disorder, Pedophilic Disorder, Coercive Sexual Sadism Disorder, and Frotteuristic Disorder

Based on the above principles, the WGSDSH has recommended the inclusion of five specifically named Paraphilic Disorder categories in the ICD-11: Exhibitionistic Disorder, Voyeuristic Disorder, Pedophilic Disorder, Coercive Sexual Sadism Disorder, and Frotteuristic Disorder. Although there were very few specific criticisms in the literature that focus on these categories in the ICD-10, there has been a considerably more active discussion in relation to the DSM-IV and DSM-5 (e.g., Blanchard, 2009, 2010a, b, 2013; Blanchard et al., 2008; First, 2010; Frances & First, 2011; Franklin, 2009; Kafka, 2010a, b; Krueger, 2010a, b; Långström, 2010), which was also considered by the WGSDSH. Some authors had suggested complete elimination of Paraphilic Disorder from diagnostic manuals (Moser & Kleinplatz, 2005), arguing that their inclusion resulted in stigmatization of those with atypical sexual interests and that these issues were best left to the legal system. This option was also considered for ICD-11.

However, the WGSDSH decided that patterns of atypical sexual arousal that involved sexual behaviors that were harmful to others by virtue of the fact that they involved actions against non-consenting individuals constituted a mental disorder according to the definition accepted for ICD-11, as well as a legitimate public health issue from WHO’s perspective. These patterns present “a clinically recognizable set of symptoms or behaviors associated in most cases with distress and with interference with personal functions” (International Advisory Group for the Revision of ICD-10 Mental and Behavioural Disorders, 2011, p. 87). Interference with functioning is generally interpreted to include causing some degree of harm to the individual or to others. Excluded from this conception of harm are the potentially negative social consequences (e.g., social exclusion) of having atypical sexual interests, so that harm emanating from such social stigmatization against those who have such interests would be excluded from the criteria for diagnosing a paraphilic disorder. Thus, the proposed definitions of ICD-11 Paraphilic Disorders have explicitly operationalized the harm associated with an atypical pattern of sexual arousal by restricting it to sexual behaviors that are harmful to self or others or which involve the individual being markedly distressed by the nature of the arousal pattern in which the distress is not simply a consequence of rejection or feared rejection of the arousal pattern by others, or where the nature of the paraphilic behavior involved significant risk of injury or death (e.g., asphyxophilia). Moreover, it was further decided that to meet the definitional requirements for a paraphilic disorder, an individual had to act on this arousal pattern or be markedly distressed by it.

The proposed named Paraphilic Disorder categories for ICD-11 include two new categories: Coercive Sexual Sadism Disorder and Frotteuristic Disorder. Coercive Sexual Sadism Disorder must be distinguished from sadomasochistic or BDSM sexual practices (bondage and discipline, dominance and submission, and sadism and masochism) characterized by consensual sexual preferences and activities. In a representative Australian study, 1.8% of sexually active individuals (2.2% of men and 1.3% of women) had engaged in BDSM sexual practices during the previous year (Richters et al., 2008). Consensual masochism and sadism as practiced in the community has not been found to be associated with poor psychological and social functioning (Krueger, 2010a, b; Wismeijer & Van Assen, 2013). For the classification to suggest that these practices are themselves constitutive of a mental disorder stigmatizes those individuals practicing them without discernible public health or clinical benefit (Cochran et al., 2014).

In contrast, Coercive Sexual Sadism Disorder, as proposed, involves the sexual arousal focused on the infliction of physical or psychological suffering on a non-consenting person as the core feature of the arousal pattern. This category is intended to provide specific forensic utility, as this pattern has been found to be an important factor among individuals who were treated in forensic institutions (Becker, Stinson, Tromp, & Messer, 2003; Berner et al., 2003; Briken, Bourget, & Dufour, 2014; Elwood, Doren, & Thornton, 2010; Packard & Levenson, 2006) and among individuals who had committed sexually motivated homicides, where the rate of sexual sadism ranged from 37 to 75% (Krueger, 2010b). This new proposed nomenclature of Coercive Sexual Sadism Disorder was selected to clearly distinguish this disorder from BDSM behaviors that are consensual and do not involve substantial harm or risk of harm. Instances of sexual masochism or sexual sadism involving marked distress or significant risk of injury or death could still be diagnosed under the category of Other Paraphilic Disorder Involving Solitary Behaviour or Consenting Individuals.

Frotteuristic Disorder, although not a named paraphilia in ICD-10, was included as a separate disorder because frotteurism, along with voyeurism and exhibitionism, has been found to be among the most common of Paraphilic Disorders reported in clinical studies (Abel et al., 1987; Bradford, Boulet, & Pawlak, 1992; Långström, 2010; Templeman & Stinnett, 1991) and in one epidemiological study (Ahlers et al., 2011) and has been reported as a significant problem in some countries (Johnson, Ostermeyer, Sikes, Nelsen, & Coverdale, 2014). This category was also continued in the DSM-5, and including it in the ICD-11 will enhance comparability across the two major diagnostic classifications.

The proposed ICD-11 Paraphilic Disorders categories (see Table 2) were recommended for inclusion based on their clear public heath utility and the need to develop and provide treatments for individuals with these disorders. While there is little information about the epidemiology of the paraphilic disorders, it is clear that a substantial proportion of those committing sexual offenses have such disorders. In a sample of 5223 sex offenders treated over a 25-year period in North America, 43% were diagnosed as being pedophiles (Maletzky, 2002), and Seto (2004) reported that “Conservatively, the prevalence of pedophilia among men who commit sexual offenses against children is around 50%, depending on the criterion used to identify pedophilia” (p. 8), and Seto suggested (2008) a prevalence rate of 1–3% for pedophilia in the male population. Eher, Rettenberger, Matthes, and Schilling (2010) found that of a sample of 114 males who were incarcerated for child molestation in the Austrian prison system, 74% had at least one paraphilic diagnosis, and 67% had a diagnosis of pedophilia.

While the use of the legal system and punishment are certainly appropriate for those who commit sexual crimes, including when such criminal behavior grows out of an underlying paraphilic disorder, identification and treatment of these disorders are important to reduce future risk (Hanson, Helmus, & Harris, 2015; Harris, Phenix, Hanson, & Thornton, 2003; Mann, Hanson, & Thornton, 2010). The WGSDSH is in no way suggesting that sexual crimes associated with paraphilic disorders be decriminalized; indeed, individuals who commit such crimes should be held accountable for their actions and criminal sanctions can be valuable and necessary in treating some individuals with paraphilic disorders. However, failure to recognize that paraphilic disorders are associated with many sexual crimes can result in lack of appropriate treatment, doing little to reduce paraphilically motivated criminal behavior. Additionally, recognition of the importance of paraphilic disorders related to the commission of some sexual crimes would support recommendations for resource allocation and appropriate structures to provide such treatment within the criminal justice system. WHO recognizes the potential impact that changes in diagnostic guidelines for paraphilic disorders may have on criminal law and forensic practice, as well as on treatment availability. For this reason, WHO has initiated legal and policy reviews in several countries in diverse regions to explore the specific implications of the proposed changes for such issues as mandatory reporting, culpability, sentencing, civil commitment, and other forensic and clinical practices.

### Creation of the Categories of Other Paraphilic Disorder Involving Non-consenting Individuals and Other Paraphilic Disorder Involving Solitary Behavior or Consenting Individuals

In addition, as shown in Table 2, the WGSDSH recommended the inclusion of the category Other Paraphilic Disorder Involving Non-Consenting Individuals in order to encompass other paraphilic arousal patterns focused on others whose age or status renders them unwilling or unable to consent that are not specifically described in any of the other named Paraphilic Disorders categories and that are not sufficiently common or well researched to include as named categories. Examples include necrophilia (sexual arousal involving corpses) and zoophilia (sexual arousal involving animals).

The WGSDSH also recommended the inclusion of the category Other Paraphilic Disorder Involving Solitary Behaviour or Consenting Individuals to describe persistent and intense patterns of atypical sexual arousal—manifested by sexual thoughts, fantasies, urges, and/or behaviors—that involve consenting adults or solitary behaviors, as long as either: (1) the person is markedly distressed by the nature of the arousal pattern and the distress is not simply a consequence of rejection or feared rejection of the arousal pattern by others or (2) the nature of the paraphilic behavior involves significant risk of injury or death (e.g., asphyxophilia or achieving sexual arousal by restriction of breathing) (Hucker, 2011). The requirement of excluding distress caused by rejection or feared rejection of the arousal pattern by others has been included to help protect against misuse of this paraphilic disorder category based on social stigmatization alone.

### Proposed Definitions and Diagnostic Guidelines for Paraphilic Disorders in ICD-11

The proposed general definition and specific essential (required) features for each Paraphilic Disorder proposed for inclusion in ICD-11 are given in Table 2. Additionally, Table 2 includes specific guidance developed in order to clearly demarcate each Paraphilic Disorder from other disorders, from normality, and from criminal behavior.

Given the atypical nature of various sexual arousal patterns and the tendency for behavior that deviates from the mainstream to be stigmatized, it is recognized that individuals with patterns of atypical sexual arousal or behavior who do not meet the diagnostic requirements for Paraphilic Disorders may experience distress associated with their sexual interests or behavior, often based on social stigmatization of negative attitudes on the part of a partner. These situations may be classified using categories that describe reasons for health encounters that are not considered to be diseases or disorders, in the ICD-11 chapter called “Factors influencing health status and contact with health services” (World Health Organization, 2015). This chapter includes a series of categories for counseling related to sexuality, including counseling related to sexual knowledge and sexual attitude, and counseling related to sexual behavior and sexual relationships.

### Continued Placement of the Paraphilic Disorders Section in the Mental and Behavioural Disorders Chapter of ICD-11

In the ICD-10, Disorders of sexual preference (F65) were included in the chapter on Mental and Behavioural Disorders. According to current proposals for the ICD-11 (World Health Organization, 2016), the categories related to sexual dysfunctions and gender identity will be moved out of the chapter on Mental and Behavioural Disorders and into a new proposed chapter on Conditions Related to Sexual Health, consistent with WGSDSH recommendations. The rationale for this reassignment for sexual dysfunctions is that the ICD-10 classification of sexual dysfunctions is based on an artificial separation of “organic” and “non-organic” sexual dysfunctions that is inconsistent with current evidence regarding the nature of sexual functioning and with current practice. Placement of sexual dysfunctions in the new chapter will permit a more integrated and clinically useful presentation of these conditions. The rationale for not conceptualizing gender incongruence as a mental disorder in ICD-11 has been described elsewhere (Drescher et al., 2012, 2016).

Although the WGSDSH considered including Paraphilic Disorders in this new chapter because of their inherently sexual nature, the WGSDSH ultimately recommended that Paraphilic Disorders remain in the Mental and Behavioural Disorders chapter because they are considered to meet the general requirements for diagnosis of a mental disorder and because their status as mental disorders is important forensically. A number of legal processes, including civil commitment, depend on their identification as mental disorders (First & Halon, 2008), and removal of the disorders from this section could result in undermining their accepted forensic usage and cast significant doubt on this entire area of case law and judicial practice. Although there are certainly legitimate controversies in this area (Janus, 2004; Zonana, 1997), changing their status as mental disorders in the ICD-11 was not considered by the WGSDSH to be a helpful or thoughtful way to address them.

## Comparison of ICD-11 Recommendations with DSM-5

In order to understand the differences between the proposed diagnostic guidelines in Paraphilic Disorders in the ICD-11 and the diagnostic criteria in the DSM-5, it is important to understand the differences between the purposes of the two classifications and the roles of the organizations responsible for them in developing international classifications for health. The 194 countries that are WHO member states agree to use the ICD as a framework for health information and reporting in order to: (1) monitor epidemics, threats to public health, and global burden of disease; (2) assess progress toward meeting public health objectives; (3) provide a framework for defining their obligations to provide free or subsidized health care to their populations; (4) facilitate access to appropriate health care services; (5) provide a basis for guidelines for care and standards of practice; and (6) facilitate research into more effective treatments and prevention strategies (International Advisory Group for the Revision of ICD-10 Mental and Behavioural Disorders, 2011). In other words, the mandate of the ICD is a pragmatic one, based on public health and clinical objectives. The guiding question underlying the development of the ICD-11 can be framed as follows: Based on the best evidence that we have available today, what health categories should the world’s global health authority tell its member states are important to track as a basis for public health reporting and as a basis for structuring clinical care, and how should those categories be defined and operationalized?

This is a substantially different set of objectives than those that underlie the American Psychiatric Association’s work on the DSM, which historically has been based on the perspectives and concerns of US psychiatrists. Inevitably, this leads to differences—and should lead to differences—between the two classifications (Kendell, 1991). The changes proposed for the Paraphilic Disorders in ICD-11 represent a major departure from its predecessor system—ICD-10—developed during the late 1980s. In contrast, the changes from DSM-IV-TR, published in 2000, compared with DSM-5 are more modest in scope. In many ways, the proposed changes for ICD-11 have brought it more in line with the DSM-5, including the removal of the requirement that the arousal patterns involved in Paraphilic Disorders be exclusive or preferential.

In a comparison of the ICD-10 and the DSM-IV, First (2009) determined that there were definitional differences without an apparent conceptual basis between the two systems in the Paraphilic Disorders categories that were compared (Fetishism, Fetishistic Transvestism, Exhibitionism, Voyeurism, and Paedophilia). The proposed ICD-11 diagnostic guidelines for Paraphilic Disorders are now conceptually closer to DSM-5 in that they require a sustained, focused, and intense pattern of sexual arousal—as manifested by persistent sexual thoughts, fantasies, urges, or behaviors and also that the individual must have acted on these thoughts, fantasies, or urges, or be markedly distressed by them. This definition parallels the A criterion in the paraphilic definitions in DSM-5, which identifies a pattern of “recurrent and intense sexual arousal” and the B criterion, which specifies that the person “has acted on these sexual urges with a nonconsenting person, or the sexual urges or fantasies cause clinically significant distress or impairment in social, occupational, or other important areas of functioning” (American Psychiatric Association, 2013).

On the other hand, there are significant differences between the proposed ICD-11 Paraphilic Disorders and DSM-5. DSM-5 includes Sexual Masochism Disorder, Fetishistic Disorder, and Transvestic Disorder as mental disorders categories, but these are not proposed as specific, named categories in ICD-11. In ICD-11, these phenomena may be diagnosed under the category Other Paraphilic Disorder Involving Solitary Behaviour or Consenting Individuals if they are associated with significant distress or significant risk of injury or death. ICD-11 also uses a duration requirement that is more flexible than the 6-month requirement for paraphilic disorder diagnoses in DSM-5, which does not appear to have specific empirical support. Instead, the ICD-11 guidelines require a clinical judgment that the arousal pattern is sustained, focused, and intense, making clear that a single instance of behavior does not meet this requirement. In keeping with the general principle for ICD that interference with social roles (e.g., family or employment) should not be used as a diagnostic requirement unless it is necessary to distinguish the disorder from normality (International Advisory Group for the Revision of ICD-10 Mental and Behavioural Disorders), this was not included as a diagnostic requirement, even though it is included relatively automatically in DSM. DSM-5 also includes a specifier to indicate whether individuals are in a controlled environment, which may be useful for forensic purposes and could be considered for ICD-11. DSM-5 also includes a qualifier for full remission, for which empirical support is very limited (First, 2014).

The evolution of the Paraphilic Disorders in the DSM and ICD has recently been reviewed by Giami (2015), who concluded that these classifications of sexual disorders reflect contemporary sexual norms and have moved from a model of pathologization or criminalization of non-reproductive sexual behaviors to a model which reflects sexual well-being and pathologizes the absence or limitation of consent in sexual relations. In this regard, the proposals for ICD-11 go further than the changes made in DSM-5, for example in the removal of disorders diagnosed based on consenting behaviors that are not in and of themselves associated with distress or functional impairment.

## Next Steps

The proposed diagnostic guidelines for Paraphilic Disorders are currently being assessed by WHO in field studies being implemented in multiple languages through WHO’s Global Clinical Practice Network (see http://gcp.network to register in any of 9 languages), the results of which will be published in due course. A detailed description of WHO’s field study methodologies for ICD-11 Mental and Behavioural Disorders has been provided by Keeley et al. (2015). The constituent categories and proposed brief glossary definitions for Paraphilic Disorders are available for public review on the WHO ICD-11 beta platform (http://apps.who.int/classifications/icd11/browse/l-m/en), and registered users may provide comments. The complete diagnostic guidelines will also be posted for review and comment on http://gcp.network (Reed et al., 2016a, b). The diagnostic guidelines will be further refined based on study results and comments received.

In addition, as mentioned above, WHO has conduced a legal and policy assessment in several countries regarding the potential impact of the proposed changes for Paraphilic Disorders as compared to ICD-10 on forensic practices and relevant national policy. While it was not possible to conduct such an assessment in all countries, participating countries were selected based on representing different global regions, languages, and legal traditions and include Brazil, Germany, India, Lebanon, Mexico, and South Africa. These assessments have now been completed and are currently being analyzed, and the results will also be published in due course.

This article also represents an effort to initiate a broader scientific discussion regarding the changes proposed. The diagnostic guidelines will also be made available for studies by other investigators, as has been the case with guidelines developed in other areas [see Hansen, Hyland, Armour, Shevlin, & Elklit (2015) for a recent example]. The results of these additional studies will also be considered by WHO in the formulation of the final version of the diagnostic guidelines prior to approval of the ICD-11 by the World Health Assembly in May 2018. It is hoped that these efforts will produce a set of guidelines that will facilitate the timely identification and effective treatment of Paraphilic Disorders that are harmful to the individual or to others while respecting the rights of individuals whose atypical sexual behavior is consensual and not harmful and will also help to clarify the interaction between clinical and legal issues in forensic and policy contexts.

## Electronic supplementary material

Below is the link to the electronic supplementary material.

Supplementary material 1 (PDF 106 kb)

Supplementary material 2 (PDF 2190 kb)

## Footnotes

1 The review process conducted by the WHO was internal.

2 The ICD-9-CM (Clinical Modification), which was in use until October 1, 2015, in the USA, is an adaptation of the ICD-9 for clinical use in the USA, originally published in 1979. U.S. National Center for Health Statistics (2011). *International Classification of Diseases, 9th revision, Clinical Modification*. Retrieved from http://www.cdc.gov/nchs/icd/icd9cm.htm.

3 Homosexuality was removed as a diagnosis in ICD-10, approved in 1990. A category of Egodystonic Sexual Orientation was retained in ICD-10, but not as a part of the Disorders of sexual preference (F65) grouping (see Cochran et al., 2014).
