# Supplementary material for: Proposals for Paraphilic Disorders in the International Classification of Diseases and Related Health Problems, Eleventh Revision (ICD-11)
Source: Arch Sex Behav. 2017 Feb 16;46(5):1529–45. doi: 10.1007/s10508-017-0944-2 (PMC5487931; doi:10.1007/s10508-017-0944-2)
Supplement: Supplementary file 1 — Supplementary material 1 (PDF 106 kb) [file 10508_2017_944_MOESM1_ESM.pdf]

ProQuest

COLUMBIA UNIVERSITY LIBRARIES

Report Information from ProQuest

December 31 2016 13:59

Access provided by Columbia University Libraries

Table of contents

1. What's in a Word? Addiction Versus Dependence in DSM-V 1

What's in a Word? Addiction Versus Dependence in DSM-V

Author: O'Brien, Charles P; Volkow, Nora; T-K, Li

ProQuest document link

Abstract:
The term "dependence" has traditionally been used to describe "physical dependence," which refers to the adaptations that result in withdrawal symptoms when drugs, such as alcohol and heroin, are discontinued.

Links: Check Columbia Libraries for fulltext

Full text:
The revision of DSM-III was the result of a great deal of work by a committee set up by APA in the 1980s. This committee worked over several years and coordinated its efforts with the World Health Organization. The result was the completely revised diagnostic classification published in 1987 known as DSM-III-R. This was an important contribution to the mental health field because it provided a clear way of defining addiction as compulsive drug-seeking behavior using criteria that turned out to have excellent interrater reliability and applicability to all forms of drug addiction. The classification was adopted with only minor changes in DSM-IV One of us (C.O.) was a member of the committee who attended every one of the committee meetings throughout the 1980s. There was good agreement among committee members as to the definition of addiction, but there was disagreement as to the label that should be used. The proponents of the term "addiction" believed that this word would convey the appropriate meaning of the compulsive drug-taking condition and would distinguish it from "physical" dependence, which is normal and can occur in anyone who takes medications that affect the CNS. Those who favored the term "dependence" felt that this was a more neutral term that could easily apply to all drugs, including alcohol and nicotine. The committee members argued that the word "addiction" was a pejorative term that would add to the stigmatization of people with substance use disorders. A vote was taken at one of the last meetings of the committee, and the word "dependence" won over "addiction" by a single vote.

Experience over the past two decades has demonstrated that this decision was a serious mistake. The term "dependence" has traditionally been used to describe "physical dependence," which refers to the adaptations that result in withdrawal symptoms when drugs, such as alcohol and heroin, are discontinued. Physical dependence is also observed with certain psychoactive medications, such as antidepressants and beta-blockers. However, the adaptations associated with drug withdrawal are distinct from the adaptations that result in addiction, which refers to the loss of control over the intense urges to take the drug even at the expense of adverse consequences. For example, research has shown that when opiates are administered to a naive animal, adaptation begins to occur after the first dose so that the second dose has a discernibly decreased effect from the first. After several days of taking the medication, abrupt cessation produces a withdrawal syndrome varying with the duration of treatment and the dose level. This is an expected pharmacological response, and although it may occur among addicts, it is quite distinct from compulsive drug-seeking behavior. This has resulted in confusion among clinicians regarding the difference between "dependence" in a DSM sense, which is really "addiction," and "dependence" as a normal physiological adaptation to repeated dosing of a medication. The result is that clinicians who see evidence of tolerance and withdrawal symptoms assume that this means addiction, and patients requiring additional pain medication are made to suffer. Similarly, pain patients in need of opiate medications may forgo proper treatment because of the fear of dependence, which is self-limiting by equating it with addiction.

The authors are now in the planning stages for DSM-V. There will be careful reviews of the criteria, but in the

case of substance use disorders, the medical world drastically needs a change in labeling. Addiction is a perfectly acceptable word. It is used by the American Society of Addiction Medicine, the American Association of Addiction Psychiatrists, the American Journal on Addictions, and the oldest journal in the field, simply known as Addiction. It is clear that any harm that might occur because of the pejorative connotation of the word "addiction" would be completely outweighed by the tremendous harm that is now being done to the patients who have had needed medication withheld because their doctors believe that they are addicted simply because they are dependent.

We urge APA to consider these patients as well as the numerous clinicians in other fields who look to DSM for the classification of mental disorders. The current labeling is not only confusing and misleading, but it contributes to suffering.

Sidebar

"Clinicians who see evidence of tolerance and withdrawal symptoms assume that this means addiction, and patients requiring additional pain medication are made to suffer."

AuthorAffiliation

CHARLES P. O'BRIEN, M.D., PH.D.

NORA VOLKOW, M.D.

T-K LI, M.D.

Address correspondence and reprint requests to Dr. O'Brien, University of Pennsylvania/VA Medical Center, 3900 Chestnut St., Philadelphia, PA 79704-6778; obrien@mail.trc.upenn.edu (e-mail).

Subject: Drug addiction; Committees; Classification; Jargon;

MeSH: Animals, Diagnosis, Differential, Diagnostic & Statistical Manual of Mental Disorders, Drug Tolerance -- physiology, Humans, Psychometrics, Substance Withdrawal Syndrome -- classification, Substance Withdrawal Syndrome -- diagnosis, Substance-Related Disorders -- classification, Psychiatric Status Rating Scales -- statistics & numerical data (major), Substance-Related Disorders -- diagnosis (major), Terminology as Topic (major)

Publication title: The American Journal of Psychiatry

Volume: 163

Issue: 5

Pages: 764-5

Number of pages: 2

Publication year: 2006

Publication date: May 2006

Year: 2006

Section: Editorial

Publisher: American Psychiatric Association

Place of publication: Washington

Country of publication: United States

Publication subject: Medical Sciences--Psychiatry And Neurology

ISSN: 0002953X

CODEN: AJPSAO

Source type: Scholarly Journals

Language of publication: English

Document type: Editorial

Accession number: 16648309

ProQuest document ID: 220489255

Document URL:
http://ezproxy.cul.columbia.edu/login?url=http://search.proquest.com/docview/220489255?accountid=10226

Copyright: Copyright American Psychiatric Association May 2006

Last updated: 2013-02-07

Database: Psychology Database,ProQuest Central,Social Science Database,Research Library

---

Contact ProQuest
Copyright © 2016 ProQuest LLC. All rights reserved. - Terms and Conditions
