# Supplementary material for: Proposals for Paraphilic Disorders in the International Classification of Diseases and Related Health Problems, Eleventh Revision (ICD-11)
Source: Arch Sex Behav. 2017 Feb 16;46(5):1529–45. doi: 10.1007/s10508-017-0944-2 (PMC5487931; doi:10.1007/s10508-017-0944-2)
Supplement: Supplementary file 2 — Supplementary material 2 (PDF 2190 kb) [file 10508_2017_944_MOESM2_ESM.pdf]

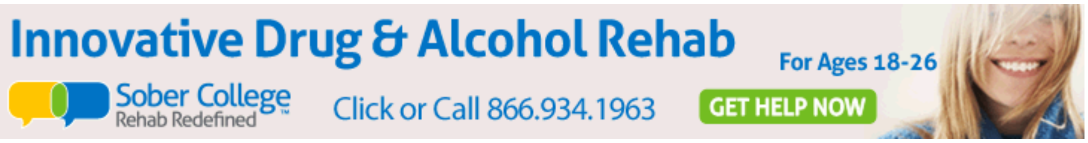

Why We Rewrote the Book on Addiction

By Charles O'Brien (/content/charles-obrien) 04/24/13

The man who led the dramatic revision of the addiction diagnosis—weathering unprecedented criticism in the process—writes that the *DSM-5* is a necessary new start.

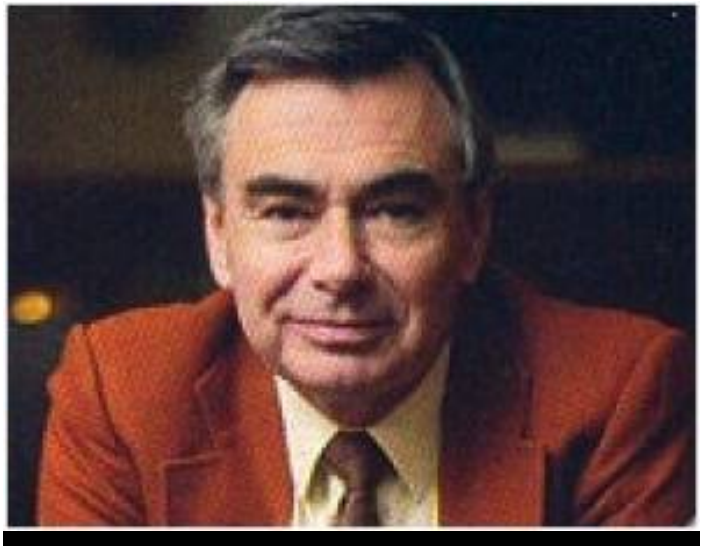

Charles O'Brien, MD, PhD - photo

Addiction is a complex, costly and debilitating condition. It exacts a heavy toll on individuals, families and society, and in this country that toll is even more acute because only a fraction of people ever get the help they need. The changes in the fifth edition of the *Diagnostic and Statistical Manual of Mental Disorders* (*DSM-5*)—I chaired the DSM Substance-Related Disorders Work Group—signal a clear break from the field's decades-old framework for identifying substance-related disorders. As beneficial as these changes will be for diagnosis, they also will have a positive impact on treatment.

This shift toward hard science is way overdue. Since the *DSM* was last published in 1994, brain imaging and other scientific advances have provided a greater understanding of the biological processes underlying addiction. We know much more about how alcohol, cocaine and other drugs activate certain areas of the brain and the neurological consequences. As a result, we now see addiction in terms of a continuum of symptoms and behaviors, not the artificial dichotomy imposed by the previous labels of "Substance Abuse" and "Substance Dependence."

The *DSM-5* resolves the longstanding debate over this abuse/dependence distinction. This debate has caused enormous confusion about the meaning of addiction among patients, the public and even clinicians.* Ending this debate is a significant accomplishment by the *DSM-5.*

The revision combines the dual classifications of abuse and dependence into a single category of "Substance Use Disorder." It defines this overarching disorder as a spectrum of symptoms that go from "mild" to "moderate" to "severe," and the criteria for each have not only been combined but strengthened. They require an additional symptom to

SEE IF YOUR INSURANCE PAYS FOR REHAB

Sponsored ⓘ

Seeking Help For *
- Select -

Insurance Company Name *

Insurance type *
- Select -

First Name * **Last Name ***

Your Email *

Best time to call

Your Phone Number *
555-555-5555
Text me

Submit Information

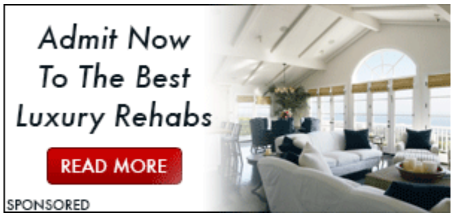

(/admit-now-luxury-rehab)

be present for a "mild" diagnosis, compared to the current criteria for substance abuse. This evidence-based change actually *raises* the requirements from the *DSM-IV* without broadening them. Additionally, the *DSM-5* criteria require that symptoms lead to clinically significant impairment or distress.

By removing the label of "dependence," it also prevents the confusion when the appropriate prescription of psychoactive medications for pain, anxiety or depression results in tolerance and withdrawal and often causes the clinician to mislabel the patient as "dependent," in the addiction sense of the word.

> *Critics say the new approach will divert resources from more serious patient cases. But that's wrongheaded—early treatment will be faster, less costly, and more successful.*

The cumulative effect of this redefinition, I believe, will advance treatment in critical ways. The characterization of the severity levels for these conditions, rather than the misguided distinction between abuse and dependence, has the potential to better direct the type and intensity of prevention and treatment services. It will inarguably improve the accuracy of treatment.

Let's start with early intervention. We've long known that the earlier we interrupt the cycle of addiction, the better the likely outcome. In the process, we may minimize or prevent serious comorbidities. The severity scale that is integral to substance use disorder will help clinicians assess individuals who are in initial ("mild") stages and focus on the most effective treatment for that point in time. This early intervention is expected to be a major public health benefit.

Such staging has not previously been used with addictions, and some critics suggest that it will divert resources from more serious patient cases. But that's wrongheaded. Why wait to act until addicts are on the brink of requiring costly hospitalizations or even transplants? We need faster, less costly, and more successful interventions and treatments—and *DSM-5* will position us for that. Our healthcare system saves $7 for every $1 spent on early care for substance use disorders, and that's another value of the "spectrum" approach.

We also foresee marked gains in the area of pain management. We've all heard too many stories of patients suffering needlessly because either they or their doctors feared—incorrectly—that a prescription for codeine or other opiate-based drugs could be a prelude to future addiction. Their misconception stemmed, once again, from the *DSM-IV*'s unfortunate construct of substance dependence, which for many people became synonymous with addiction.

Yet studies have shown that dependence can be the body's normal response to a chemical. Clinically, the word describes only physical symptoms, as evidence of increasing levels of tolerance, not any psychological response involving compulsive, pathological behavior. The latter, of course, is what's key to actual addiction. We expect that the new unified approach to diagnosis will clarify the distinction. Tremendous harm has been done to some patients who have had legitimate, needed medication withheld. But no more, we hope.

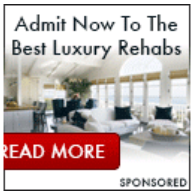

SOBER SYSTEMS RECOVERY 2.0

FEATURED CENTERS MORE >

01 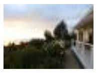 (/content/cliffside-malibu0005)

Cliffside Malibu (/content/cliffsi malibu0005)

★★★★★

The "beach-house-relaxed" **Cliffside Malibu** claims to provide an oasis for recovering addicts and alcoholics. And that's just what you'll get—if you've got the cash.

02 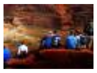 (/content/back2basics-outdoor-adventures0010)

Back2Basics Outdoor Adventures (/content/back2 outdoor-adventures0010

★★★★

This **Arizona rehab** prescribes high doses of AA meetings and backpacking for young guys who not only need to get sober, but also learn the basics (think cooking and cleaning) of living in the real world.

03 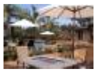 (/content/aton-center-san-diego-rehab00430)

AToN Center (/content/aton-center-san-diego-rehab00430)

★★★★½

Whether you're interested in the 12 Steps, SMART Recovery, or holistic treatments, this luxurious, appealing and commendable 4.5 star **San Diego rehab** has a program for you.

As its diagnostic starting point, the *DSM-5* addresses each specific substance as a separate disorder ("cannabis-use disorder," "alcohol-use disorder," etc.), with the severity of the disorder depending on how many symptoms a person has. Detractors continue to argue that the thresholds for diagnoses have been lowered and that that will lead to increases in prevalence. Their claim is hard to support based on numbers alone. Nearly a dozen symptoms now must be present for the most serious diagnosis.

In the Work Group's due diligence, we looked at the impact these changes could have on the prevalence of substance use disorders. In a secondary analysis of data from 43,000 randomly selected Americans, no significant change in prevalence was indicated. Several smaller studies supported this finding.

Collectively, the definitions and criteria debuting in May with the new manual's publication offer us a more comprehensive and multidimensional model of care for any substance use disorder. They represent essential progress in diagnosis, and I'm convinced that they will immediately begin to influence treatment. But they're hardly the final word. For the sake of the millions of people struggling to overcome an addiction, we must push much further ahead.

Charles O'Brien, MD, PhD, is a world-renowned specialist in addiction psychiatry. He served as the chair of the DSM Substance-Related Disorders Work Group for the DSM-5. *He is the vice-chair of psychiatry at the University of Pennsylvania, and the director of its Center for Studies in Addiction. He was elected to the Institute of Medicine of the National Academy of Sciences in 1991, and has advised the US government on drug policy for decades.*

*In 2006, Nora Volkow, MD, the director of the National Institute on Drug Abuse, T.K. Li, MD, then-head of the National Institute on Alcohol Abuse and Alcoholism, and O'Brien co-authored an editorial (http://ajp.psychiatryonline.org/article.aspx?articleid=97366) in the May *American Journal of Psychiatry* stating unequivocally that "experience over the past two decades has demonstrated that the decision [by the *DSM-IV* to include "dependence"] was a serious mistake."

Please read our comment policy (/content/fix-comment-policy). - *The Fix*

10 Comments (https://www.thefix.com/content/dsm-5-substance-use-disorder-addiction-early-intervetion8615)

TAGS: DSM-5 (/tags/dsm-5)
DSM Substance-Related Disorders Work Group (/tags/dsm-substance-related-disorders-work-group)
Substance Abuse (/tags/substance-abuse)
substance dependence (/tags/substance-dependence)
Nora Volkow (/tags/nora-volkow)
National Institute on Drug Abuse (/tags/national-institute-drug-abuse-0)
T-K Li (/tags/t-k-li)
National Institute on Alcohol Abuse and Alcoholism (/tags/national-institute-alcohol-abuse-and-alcoholism)
Journal of Psychiatry (/tags/journal-psychiatry)
substance use disorder (/tags/substance-use-disorder)
Professional Voices (/tags/professional-voices) Features (/features)
Charles O'Brien (/content/charles-obrien)

You May Also Enjoy

04 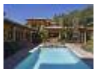 (/content/reflections-marin-county-rehab00340)

Reflections (/content/reflec marin-county-rehab00340)

★★★★

This exclusive **Northern California rehab** is all about client choice—as well as golf outings, Buddhist field trips and keeping up with the office.

05 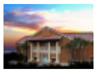 (/content/gulf-breeze-recovery-0)

Gulf Breeze Recovery (/content/gulf-breeze-recovery-0)

★★★★½

An alternative to 12-step programs, Gulf Breeze Recovery offers panoramic ocean views in a caring and therapeutic environment. Housing up to 26 residential guests, this luxury facility offers a choice of semi-private or private rooms with or without ocean views.

06 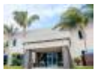 (/content/morningside-recovery00378)

Morningside Recovery (/content/morni recovery00378)

★★★

Morningside Recovery's flexible and "real world" model encourages the acquisition of life skills while giving clients a strong and individualized therapeutic foundation.

07 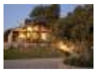 (/content/newport-academy0001)

Newport Academy (/content/newp academy0001)

★★★★★

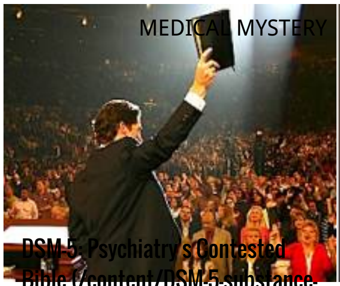
MEDICAL MYSTERY

DSM-5: Psychiatry's Contested Bible (/content/DSM-5-substance-use-disorder-cost-effective8083)

(/content/DSM-5-substance-use-disorder-cost-effective8083)

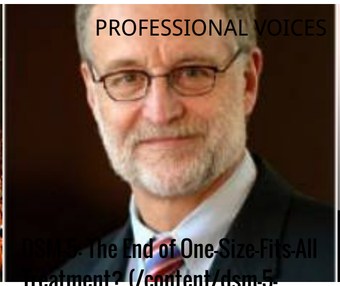
PROFESSIONAL VOICES

DSM-5: The End of One-Size-Fits-All Treatment? (/content/dsm-5-substance-use-disorder-individualized-treatment8614)

(/content/dsm-5-substance-use-disorder-individualized-treatment8614)

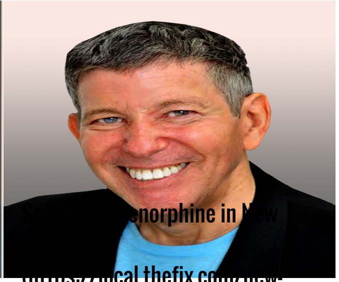

[illegible]norphine in [illegible] (https://local.thefix.com/new-york/?category_name=buprenorphine)

(https://local.thefix.com/new-york/?category_name=buprenorphine)

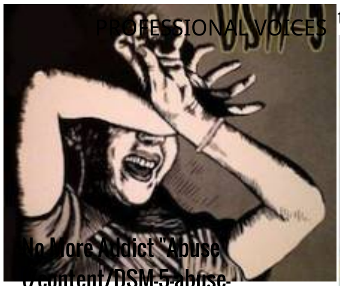
PROFESSIONAL VOICES

No More Addict "Abuse" (/content/DSM-5-abuse-dependence-substance-use-disorder8423)

(/content/DSM-5-abuse-dependence-substance-use-disorder8423)

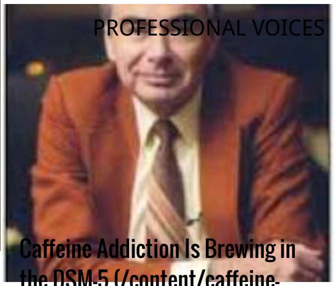
PROFESSIONAL VOICES

Caffeine Addiction Is Brewing in the DSM-5 (/content/caffeine-intoxication-withdrawal-coffee-srimulant8500)

(/content/caffeine-intoxication-withdrawal-coffee-srimulant8500)

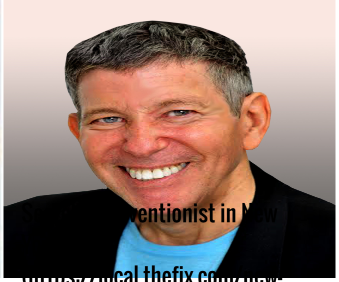

[illegible]entionist in [illegible] (https://local.thefix.com/new-york/?category_name=interventionist)

(https://local.thefix.com/new-york/?category_name=interventionist)

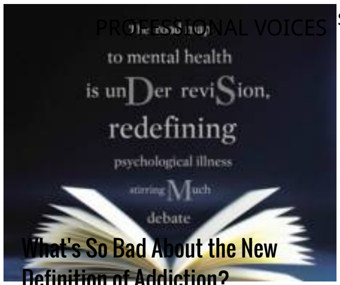

PROFESSIONAL VOICES

What's So Bad About the New Definition of Addiction? (/content/professional-voices-dsm-5-revisions-controversy8529)

(/content/professional-voices-dsm-5-revisions-controversy8529)

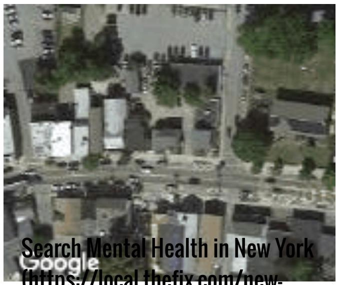

Search Mental Health in New York (https://local.thefix.com/new-york/?category_name=mental-health)

(https://local.thefix.com/new-york/?category_name=mental-health)

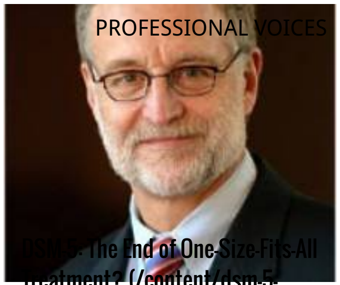
PROFESSIONAL VOICES

DSM-5: The End of One-Size-Fits-All Treatment? (/content/dsm-5-substance-use-disorder-individualized-treatment8614)

(/content/dsm-5-substance-use-disorder-individualized-treatment8614)

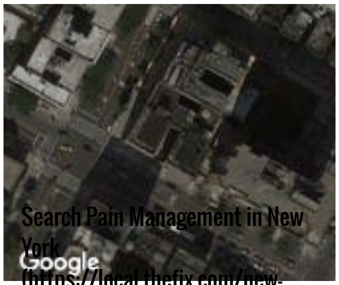

Search Pain Management in New York (https://local.thefix.com/new-york/?category_name=pain-management)

(https://local.thefix.com/new-york/?category_name=pain-management)

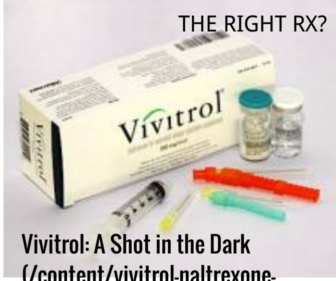

THE RIGHT RX?

Vivitrol: A Shot in the Dark (/content/vivitrol-naltrexone-addiction-craving8033)

(/content/vivitrol-naltrexone-addiction-craving8033)

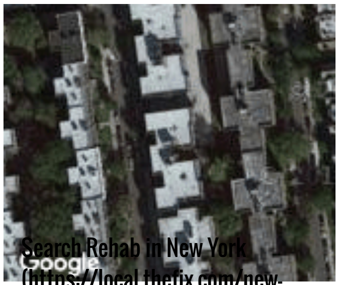

Search Rehab in New York (https://local.thefix.com/new-york/?category_name=rehabs)

(https://local.thefix.com/new-york/?category_name=rehabs)

This SoCal rehab fosters a regimented but respectful recovery environment, where teens learn how to live sober through plenty of 12-step meetings and life-skills classes—not to mention "equine-assisted psychotherapy" and mixed martial arts.

08 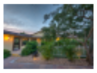 (/amethyst-recovery-center)

Amethyst Recovery Center (/amethyst-recovery-center)

★★★★

With 12-step and faith-based options, Amethyst gets high praise for its staff (in recovery themselves) and the camaraderie that develops among the clients.

09 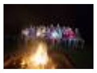 (/content/discovery-place)

Discovery Place (/content/disco[illegible] place)

★★★★½

This 12-step based non-profit Tennessee facility for men offers meetings, groups, camaraderie, and boasts an impressive success rate.

010 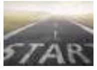 (/how-choose-best-rehab)

How to Choose The Best Rehab (/how-choose-best-rehab)

★★★★★

What you need to know when choosing an addiction treatment center.

10 Comments (https://www.thefix.com/content/dsm-5-substance-use-disorder-addiction-early-intervetion8615)

0

Recommend Be the first of your friends to recommend this.

89

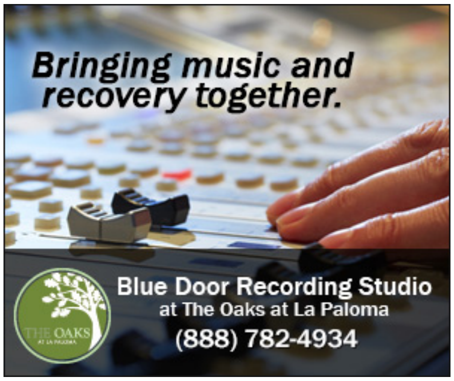

READER FORUMS

MORE >

207 (/forum/aa/medication-assisted-treatment-meetings#disqus_thread) replies

Medication-Assisted Treatment in Meetings (/forum/aa/medication-assisted-treatment-meetings)

55 (/forum/general-discussion/aa-under-attack#disqus_thread) replies

AA under attack (/forum/general-discussion/aa-under-attack)

31 (/forum/celebrities/do-celebrities-have-responsibility-come-out-sober-closet#disqus_thread) replies

Do celebrities have a responsibility to come out of the sober closet? (/forum/celebrities/do-celebrities-have-responsibility-come-out-sober-closet)

20 (/forum/legalization-marijuana/big-marijuana-next-big-tobacco#disqus_thread) replies

Is Big Marijuana The Next Big Tobacco? (/forum/legalization-marijuana/big-marijuana-next-big-tobacco)

Login to Post a Topic (/hybridauth/window/Disqus?destination=forum&destination_error=forum)

MOST POPULAR

1. 5 Characteristics of Adult Children of Alcoholics (http://www.thefix.com/content/5-characteristics-adult-children-alcoholics)
2. Suboxone Addiction: Symptoms, Side Effects, Withdrawal, Detox, and Rehab (http://www.thefix.com/content/suboxon addiction)
3. Suicidal in Sobriety (http://www.thefix.com/content/russian-roullete)
4. AA vs. NA—What's the Difference? (http://www.thefix.com/content/aa-vs-na)
5. Kickin' Kratom (http://www.thefix.com/content/kickin%E kratom)

LIVING SOBER

MORE >

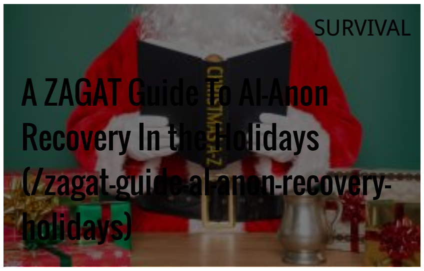

SURVIVAL

A ZAGAT Guide To Al-Anon Recovery In the Holidays (/zagat-guide-al-anon-recovery-holidays)

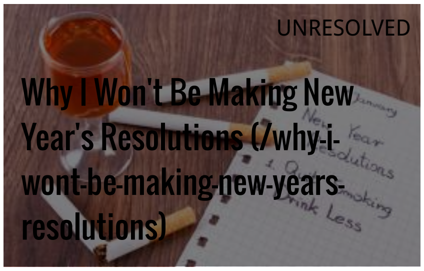

UNRESOLVED

Why I Won't Be Making New Year's Resolutions (/why-i-wont-be-making-new-years-resolutions)

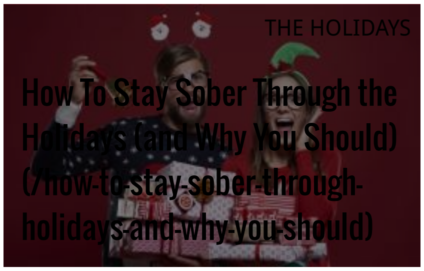

THE HOLIDAYS

How To Stay Sober Through the Holidays (and Why You Should) (/how-to-stay-sober-through-holidays-and-why-you-should)

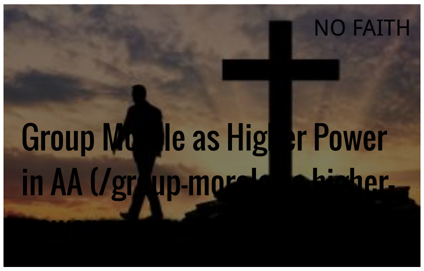

Group M[illegible]le as Hig[illegible]r Power in AA (/gr[illegible]up-mor[illegible]

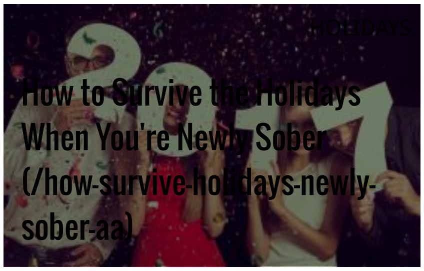

How to Survive th[illegible] Holidays When You're Newl[illegible] Sober (/how-survive-ho[illegible]days-newly-sober-aa)

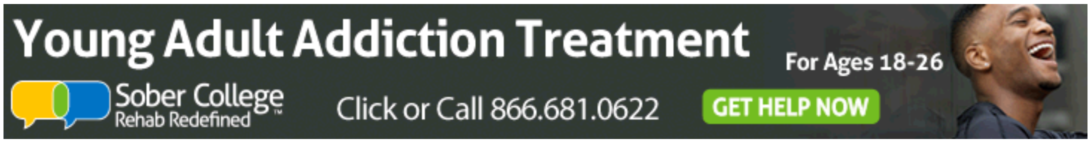

Rehab Directory (https://local.thefix.com/) | Treatment Info (/substance-abuse-resources-rehab-detox) | Insurance Info (/how-use-insurance-benefits-addiction-treatment) | Contact Us (/content/contact-us) | Advertise on The Fix (/content/advertise-fix) | Privacy Policy (/content/privacy-policy) | Terms & Conditions (/content/terms-conditions-1)

(http://[illegible])
